# Attenuating ischemia/reperfusion injury in rat cardiac transplantation by intracoronary infusion with siRNA cocktail solution

**DOI:** 10.1042/BSR20193937

**Published:** 2020-08-04

**Authors:** Bo Yang, Jin Wang, Yuanyuan Zhao, Wu Duan, Chen Dai, Zhenyi Han, Meixi Wang, Bo Zhang, Lai Wei, Zhishui Chen, Dong Chen

**Affiliations:** 1 Institute of Organ Transplantation, Tongji Hospital, Tongji Medical College, Huazhong University of Science and Technology, Wuhan 430030, China; 2 Key Laboratory of Organ Transplantation, Ministry of Education, Wuhan 430030, China; 3 NHC Key Laboratory of Organ Transplantation, Wuhan 430030, China; 4 Key Laboratory of Organ Transplantation, Chinese Academy of Medical Sciences, Wuhan 430030, China; 5 Division of Endocrinology, Department of Internal Medicine, Qilu Hospital of Shandong University, Jinan, China; 6 Department of Endocrinology, Tongji Hospital, Tongji Medical College, Huazhong University of Science and Technology, Wuhan 430030, China

**Keywords:** Cardiac transplantation, Graft survival, Intracoronary infusion, Oxidative stress damage, Small interfering RNAs

## Abstract

Tumor necrosis factor-α (TNF-α), caspase-8, and complement component 5a receptor (C5aR) are known to play a crucial role in the myocardial ischemia/reperfusion (I/R) injury in cardiac transplantation. We hypothesized that the intracoronary infusion of TNF-α, caspase-8, and C5aR small interfering RNAs (siRNA) would protect cardiac allograft function and improve graft survival from I/R injury-induced organ failure. I/R injury of cardiac allograft was induced by syngeneic rat cardiac transplantation, in which the transplanted hearts were infused with saline or different amounts of siRNA cocktail solution targeting TNF-α, caspase-8, and C5aR via coronary arteries, and subsequently subjected to 18 h of preservation at 4°C in histidine–tryptophan–ketoglutarate (HTK) solution. The effects of siRNA cocktail solution on prolonged cold I/R injury were determined by assessing graft survival, histopathological changes, myeloperoxidase (MPO) activity, and malondialdehyde (MDA) concentration. The perfused siRNA cocktail solution successfully knocked down the expression of TNF-α, caspase-8, and C5aR *in vitro* and *in vivo*. Approximately 91.7% of control hearts that underwent 18 h of cold ischemia ceased their function after transplantation; however, 87.5% of cardiac allografts from the highest dose siRNA cocktail solution-pretreated hearts survived >14 days and exhibited minimal histological changes, with minimal cellular infiltration, interstitial edema, and inflammation and maximal reduced MPO activity and MDA concentration in the cardiac allograft. We demonstrated the feasibility and efficiency of infusion of TNF-α, caspase-8, and C5aR siRNA via the intracoronary route as a promising strategy for gene silencing against I/R injury in cardiac transplantation.

## Introduction

Organ transplantation is a common therapy for end-stage organ failure. However, transplantation requires cold storage of organs and warm blood transported back to them. Ischemia/reperfusion (I/R) injury caused by this process is still a serious problem affecting the outcome of transplantation [1]. I/R injury usually results in early graft dysfunction or primary nonfunction. Furthermore, I/R injury plays an important role in the development of chronic graft failure [2,3]. I/R injury involves a series of cascade reactions, such as hypoxia, acidosis, and tissue damage, which lead to many post-transplantation events, including cytokines, chemokines, up-regulation of complement, and cell apoptosis [4–6].

Tumor necrosis factor-α (TNF-α), caspase-8, and complement component 5a receptor (C5aR) are known to play a crucial role in the development of myocardial I/R injury. TNF-α is a cytokine that may initiate the reperfusion-dependent cytokine cascade, induce the expression of adhesion molecules and chemokines, and promote leukocyte infiltration, thus, leading to myocardial injury [7,8]. The importance of caspase is that it functions as an intracellular cysteine protease that mediates cell death and inflammation. Caspase-8 is the main mediator of apoptosis and necrotic cell death. In cases of caspase-8 deficiency or its inhibition, ischemic brain, heart, and kidney injuries can be effectively prevented [9–11]. Myocardial ischemia and reperfusion can be mediated by the activation of the complement system. C5aR is an important pathogenic factor that can mediate myocardial cell injury, the blocking of which may be a useful therapeutic mechanism to prevent myocardial I/R injury. Blocking C5aR was seen to significantly reduce microvascular permeability in ischemic myocardium and leukocyte adhesion to coronary artery endothelium [12,13].

Gene transfer technology has increasingly higher applications in manipulating gene expression. RNA interference, using double-stranded small interfering RNA (siRNA), can allow gene-specific silencing by delivering highly homologous RNA to cells [14]. In *in vivo* experiments, gene transfer to the donor heart has been successfully achieved by direct myocardial injection and intracoronary infusion. Since direct injection results in uneven distribution of transgene expression and local inflammation, the latter method is preferable for clinical applications [15,16]. In the present study, we transfected the donor heart by intracoronary infusion of an siRNA cocktail solution targeting TNF-α, caspase-8, and C5aR to reduce cardiomyocyte sensitivity to I/R injury during cardiac transplantation in rats. It is our belief that implementation of this will improve the quality of the preserved cardiac allograft and increase its survival.

## Materials and methods

### Animals

Lewis rats, male, weighing 200–250  g, were purchased from Beijing HFK Bioscience Co., Ltd. (Beijing, China), maintained in the Animal Facility of Tongji hospital, Tongji Medical College, Huazhong University of Science and Technology under standard conditions, and allowed to drink water and eat rodent chow *ad libitum*. The rats were under intraperitoneally administered ketamine (100 mg/kg) and xylazine (20 mg/kg) anesthesia and were killed by exsanguination from abdominal aorta; every effort was taken to minimize any suffering of the animals throughout the study. All procedures involving animal use in the present study were performed and monitored in accordance with the guidelines of the Chinese Council on Animal Care and approved by the Institutional Animal Care and Use Committee of the Tongji Medical College, Huazhong University of Science and Technology.

### siRNA synthesis

Multiple siRNA sequences were designed and synthesized without any modifications by Guangzhou RiboBio Co., Ltd. (Guangzhou, China). The sense sequences are as follows (5′–3′): TNF-α-1: TGTTTAGACAACTGGCTGC; TNF-α-2: GCAGCCAGTTGTCTAAACA; Caspase-8-1: TTAAAGGTCTTACTCAGAGCC; Caspase-8-2: GGCTCTGAGTAAGACCTTTAA; C5aR-1: TGAAACTCTTGCTGTCCCTGC; C5aR-2: GCAGGGACAGCAAGAGTTTCA.

### Heterotopic cardiac transplantation

Cardiac transplantations were performed using the heterotopic cardiac transplantation model described by Ono et al. [17]. In brief, the transfected donor heart was procured by the procedure described above and then was transplanted into the abdomen of another Lewis rat of the same strain. The heterotopic heart transplantation was performed by anastomosing the descending aorta to the abdominal aorta and the pulmonary artery to the inferior caval vein in an end-to-side fashion. The ischemic time was 40 ± 5 min. Upon re-establishment of blood flow, transplanted heart resumed spontaneous contractions in sinus rhythm and was free of gross surgical injury at the time of closure.

### Animal grouping and sample procurement

Rat heterotopic cardiac transplantation was conducted in four groups: group 1 (*n*=12), prospective cardiac donor was intracoronary infused with 0.5 ml of 0.9% saline when the heart was harvested; group 2 (*n*=6), 10 μg × TNF-α, caspase-8, and C5aR siRNA mixture was delivered through intracoronary infusion on harvesting the donor heart; group 3 (*n*=8), 50 μg × TNF-α, caspase-8, and C5aR siRNA mixture was delivered through intracoronary infusion on harvesting the donor heart; and group 4 (*n*=8), 100 μg × TNF-α, caspase-8, and C5aR siRNA mixture was delivered through intracoronary infusion on harvesting the donor heart. After 18 h of storage at 4°C in HKT solution, the hearts of these four groups were transplanted into the syngeneic recipients. The recipient rats were killed on the second day after transplantation, and cardiac allograft tissue samples were collected and stored for future processing.

### Quantitative real-time PCR

Total RNA was isolated from cells and heart tissue using TRIzol following the manufacturer’s instructions (Takara, Japan). Total RNA (1 µg) was reverse transcribed into cDNA using PrimeScript RT Master Mix (Takara, Japan) as indicated. Real-time PCR amplification was performed using the ABI 7500 system (Applied Biosystems, U.S.A.). The following primers were used: TNF-α, 5′ TGATCCGAGATGTGGAACTGG 3′ (sense) and 5′ CTCCTCCGCTTGGTGGTTT 3′ (antisense); Caspase-8, 5′ TGTTTTGGATGAGGTGAC 3′ (sense) and 5′ TTGCTGAGTTTGGGTATG 3′ (antisense); C5aR, 5′ TCACCACAGAGCCCAGGAGAA 3′ (sense) and 5′ AGAAACCAAATGGCGTTGACAG 3′ (antisense); GAPDH, 5′ CTGGAGAAACCTGCCAAGTATG 3′ (sense) and 5′ GGTGGAAGAATGGGAGTTGCT 3′ (antisense). The PCR was carried out at 95°C for 30 s, followed by 45 cycles at 95°C for 5 s, 60°C for 34 s, and 95°C for 15 s. The amount of mRNA of each gene was normalized with GAPDH and the relative expression level was calculated by the 2^−ΔΔ*C*_T_^ method [18].

### Histopathological studies

The hearts were removed and preserved in neutral buffered formalin for histopathological examination. The heart slices were then embedded in paraffin. Sectioning and Hematoxylin–Eosin (HE) staining was performed according to routine histological procedures. The slides were examined under a light microscope (Olympus BX51, Olympus Corp, Tokyo, Japan).

### Immunohistochemistry for detection of myeloperoxidase

The myeloperoxidase (MPO) activity in the heart was assessed using an anti-MPO kit by immunohistochemistry. The analysis was conducted by two pathologists who were blinded to the objective of the study. Staining of neutrophil cytoplasmic MPO was evaluated and the results were expressed as the percentage of 1000 cytoplasmic MPO-positive neutrophils counted in the same section.

### Determination of lipid peroxidation

The determination of malondialdehyde (MDA) is widely used as an indicator of lipid peroxidation. The MDA concentration was calculated using a commercial assay kit (Bioxytech MDA-586, OxisResearch, U.S.A.) according to the manufacturer’s instructions [19].

### Statistical analysis

All data are expressed as mean ± standard deviation (SD). The data were analyzed using a one-way analysis of variance followed by the least squares difference test (assuming equal variances) or Tamhane’s T2 test (without the assumption of equal variances). Comparisons between groups were performed with the two-tailed Student’s *t* test. To compare graft survival, the Mann–Whitney U test was used. All differences were considered to be statistically significant at *P*-values <0.05.

## Results

### *In vitro* siRNA cocktail solution targeting TNF-α, Caspase-8, and C5aR

**Figure 1:**
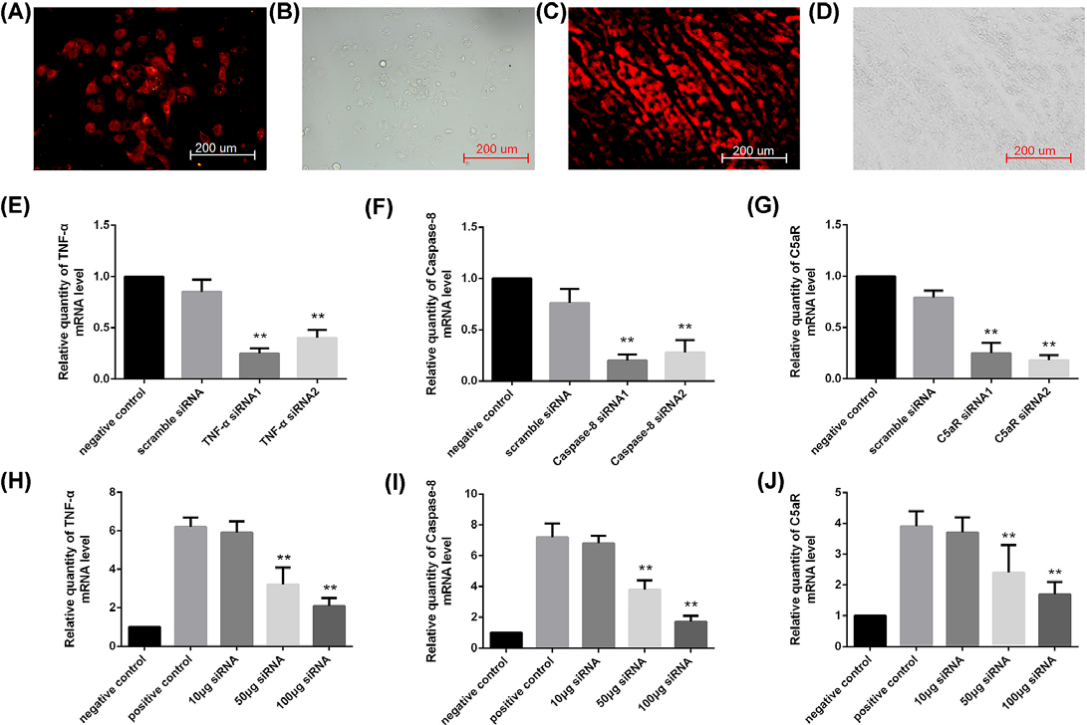
*In vitro* and *in vivo* gene silencing of TNF-α, caspase-8, and C5aR by siRNA cocktail solution (**A,B**) Dark and Bright field images of H9c2 cells after Cy3-labeled siRNA cocktail solution transfection by fluorescence microscopy, respectively. (**C,D**) Fluorescence microscopy photographs of the collected rat hearts 2 days after Cy3-labeled siRNA cocktail solution injection through the tail vein (fluorescence field: C, bright field: D). (**E**–**J**) Knock down of endogenous expression measured by qRT-PCR for TNF-α, caspase-8, and C5aR *in vitro* (E–G) and *in vivo* (H–J). Densitometric analysis was performed and the relative expression of each band was normalized to GAPDH. The data are shown as the mean ± SD (**, *P*<0.01).

The siRNA fragments were designed against rat TNF-α, caspase-8, or C5aR. Before the siRNA intracoronary delivery to rat heart, we examined whether siRNA could suppress TNF-α, caspase-8, or C5aR expression efficiently. Rat myocardial cell H9c2 cells were transfected with TNF-α, caspase-8, or C5aR siRNA to evaluate the possible off-target effects. In order to confirm transfection efficacy, siRNA was fluorescently labeled with Cy3 conjugated at the 5′ end. The transfection efficiencies *in vitro* are shown in Figure 1A,B (>90%). Relative amounts of TNF-α, caspase-8, or C5aR were measured by quantitative real-time PCR (qRT-PCR), normalized to GAPDH. As shown in Figure 1E–G, TNF-α, caspase-8, or C5aR sequence specific siRNA potently suppressed the expression of target gene mRNA (>70%). Transfection with scrambled siRNA resulted in no significant alteration of target gene RNA expression compared with non-transfected H9c2 cells. In addition, TNF-α siRNA-1, caspase-8 siRNA-1, or C5aR siRNA-2 have a higher interference effect than the other designed siRNA fragments, so they were used as an siRNA cocktail ingredient in the following experiments.

### siRNA cocktail solution knocks down the expression of TNF-α, caspase-8, and C5aR *in vivo*

To demonstrate the expression of TNF-α, caspase-8, or C5aR siRNA in the myocardial tissue after intracoronary infusion, the cardiac sections were observed for fluorescence of Cy3-labeled siRNA at day 2 after transplantation (red fluorescence). Compared with the control-transfected cardiac allografts, siRNA cocktail solution pretreated cardiac allografts demonstrated that the TNF-α, caspase-8, or C5aR siRNA were globally distributed throughout the myocardium after intracoronary infusion of the siRNA cocktail solution (Figure 1C,D).

We further investigated the suppressive effect of siRNA cocktail solution targeting TNF-α, caspase-8, or C5aR *in vivo*. At 48 h after transplantation, we analyzed the levels of TNF-α, caspase-8, and C5aR mRNA in the cardiac allograft by qRT-PCR. TNF-α, caspase-8, and C5aR mRNA levels of intracoronary infused heart treated with varying amounts of siRNA cocktail solution were inhibited differently: 50 and 100 μg siRNA cocktail solution treatment significantly reduced the TNF-α, caspase-8, and C5aR expression compared with those treated with saline (positive control). Furthermore, a reduction of more than 50% in TNF-α, caspase-8, and C5aR mRNA concentration was noted in the rats treated with 100 μg siRNA cocktail solution intracoronary infusion compared with that in the positive control rats (Figure 1H–J). Taken together, inhibition of TNF-α, caspase-8, and C5aR mRNA was siRNA dose-dependent effect.

### The minimal histological changes in high-dose siRNA cocktail solution-treated cardiac allografts

**Figure 2:**
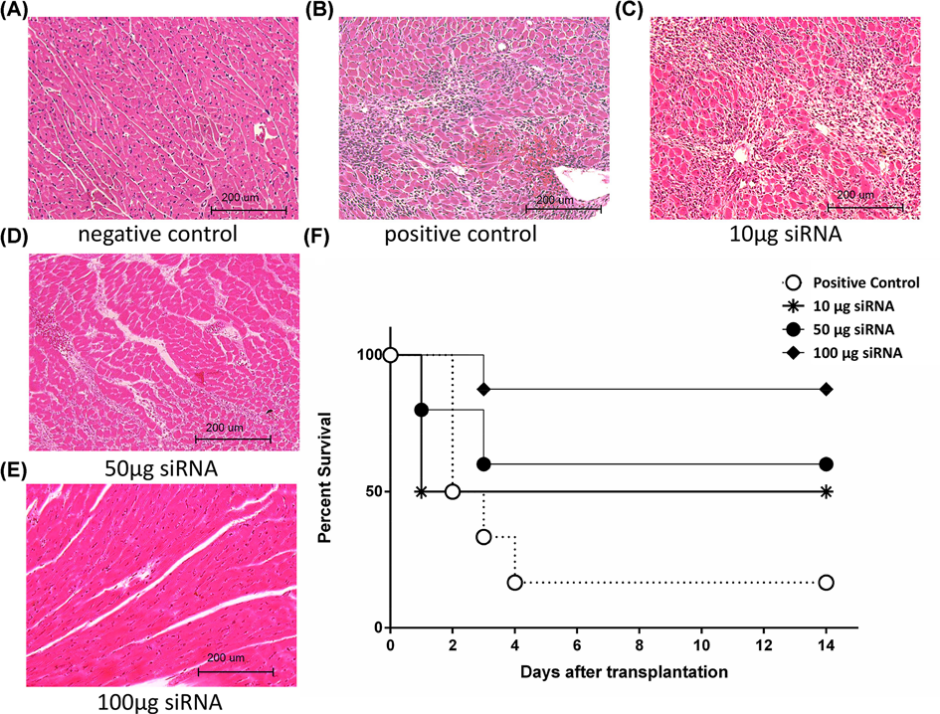
Intracoronary infusion of siRNA cocktail solution reduces the histological changes and prolongs survival of cardiac allografts after cold I/R injury H&E staining for cardiac allografts harvested at 48 h after transplantation. (**A**) Native heart (negative control); (**B**) cardiac graft treated with saline (positive control); (**C–E**) cardiac graft treated with 10, 50, and 100 μg siRNA cocktail solution (magnification: 400×). (**F**) Overall survival of the cardiac allografts was 8.3, 16.6, 37.5, and 87.5% in positive control, 10, 50, and 100 μg siRNA cocktail solution treatment group, respectively. Significant prolongation of graft survival was achieved with 100 μg siRNA cocktail solution treatment compared with positive control and 10 μg (*P*<0.01) or 50 μg siRNA cocktail solution treatment (*P*<0.05). When compared with the positive control, 50 μg siRNA treatment significantly improved graft survival (*P*<0.05), while 10 μg siRNA treatment did not (*P*>0.05).

We carried out the histological examination of standard H&E-stained sections to evaluate the severity of I/R injury by means of siRNA cocktail solution intracoronary infusion for each group. siRNA (3 × 100 μg) intracoronary infused cardiac allografts showed minimal histological changes with minimal cellular infiltration, interstitial edema, and inflammation compared with other groups. Dense interstitial infiltration, consisting primarily of erythrocytes, neutrophils, and mononuclear cells in the perivascular space and between myotubes, was observed in the control and 3 × 10 μg siRNA group (Figure 2A–E).

### Intracoronary infusion of siRNA cocktail solution prolongs survival of cardiac allografts after cold I/R injury

Eleven out of twelve (91.7%) of control hearts that underwent 18 h of cold ischemia ceased to function after transplantation into syngeneic Lewis rats (group 1). Only one cardiac allograft survived >14 days in this group. Five out of six (84.4%) of the cardiac allografts stopped beating after TNF-α, caspase-8, and C5aR siRNA (3 × 10 μg) therapy (group 2). However, TNF-α, caspase-8, and C5aR siRNA (3 × 50 μg) improved the survival of three out of eight cardiac allografts to >14 days (group 3). In marked contrast, 87.5% (7/8) of cardiac allografts from TNF-α, caspase-8, and C5aR siRNA (3 × 100 μg) pretreated donors survived >14 days (group 4). The survival of cardiac allografts was dependent on the dose of TNF-α, caspase-8, and C5aR siRNA intracoronary infusion (Figure 2F).

### The high-dose siRNA cocktail solution reduces MPO activity and MDA concentration in the cardiac allografts

**Figure 3:**
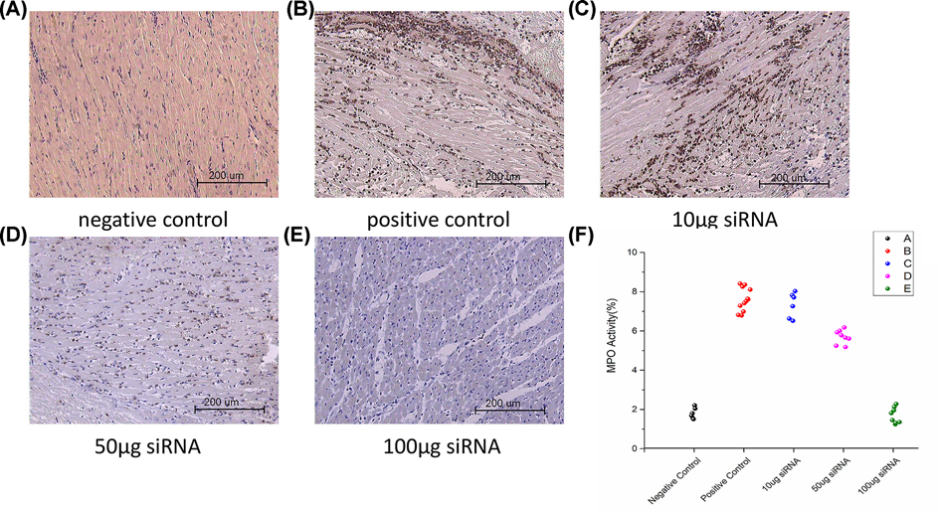
The high-dose siRNA cocktail solution reduces MPO activity in the cardiac allografts The MPO activity in cardiac allografts was measured by immunohistochemical analysis. (**A**) Normal heart (negative control); (**B**) saline intracoronary infusion at harvest (positive control); (**C**–**E**) 10, 50, or 100 μg siRNA cocktail solution intracoronary infusion at harvest; (**F**) statistics of MPO activity in the cardiac grafts without or with different doses of siRNA cocktail solution. The data are shown as mean ± SD (*P*<0.01 vs normal heart; *P*<0.01 vs positive control).

There were significant differences in MPO activity among the different amounts of siRNA cocktail solution-treated groups. Figure 3 showed the effects of TNF-α, caspase-8, and C5aR siRNA on cardiac MPO activity. The percentage of neutrophils with cytoplasm that stained positive for MPO in the 50 and 100 μg siRNA-treated rats were significantly lower than that in those administered with saline (positive control) and 10 μg siRNA. TNF-α, caspase-8, and C5aR siRNA dose-dependently reduced the increase in cardiac MPO activity. The increase in cardiac MPO activity correlated with the increase in leukocyte infiltration of transplanted cardiac tissue.

MDA, an end-product of lipid peroxidation, was measured in the cardiac graft tissue to assess the severity of oxidative damage after cold I/R injury. For both saline and siRNA cocktail solution intracoronary infused cardiac allografts, MDA levels in the transplanted cardiac tissue after 18 h of preservation in histidine–tryptophan–ketoglutarate (HTK) solution were significantly increased compared with those in the native cardiac tissue. Although the MDA level was not significantly altered by 10 μg siRNA, it decreased significantly in the transplanted cardiac tissue with increase in the dose of siRNA (Table 1).

**Table 1:** Effect of siRNA cocktail solution treatment on MDA levels in the cardiac allografts

Treatment	*n*	MDA level (μM) mean ± SD
Native heart (negative control)	6	1.8 ± 0.3
Saline intracoronary infusion at harvest (positive control)	12	7.6 ± 0.8^1^
10 μg siRNA intracoronary infusion at harvest	6	7.3 ± 0.8^1^
50 μg siRNA intracoronary infusion at harvest	8	5.7 ± 0.5^2^
100 μg siRNA intracoronary infusion at harvest	8	1.7 ± 0.6^2^

Significantly higher compared with the negative control group (^1^*P*<0.01).

Significantly lower compared with the positive control group (^2^*P*<0.01).

## Discussion

In the present study, we have demonstrated that intracoronary infusion of siRNA cocktail solution can achieve TNF-α, caspase-8, and C5aR gene silence in rat cardiac allografts and result in myocardial protection after I/R injury induced by cold preservation. It has also been shown that this myocardial protective effect of TNF-α, caspase-8, and C5aR gene silencing is associated with reduced inflammatory response, as evaluated by histological studies, MPO activity assays, and MDA concentration, as well as with improved cardiac allografts survival.

We believe that this protective effect of TNF-α, caspase-8, and C5aR gene silencing, as shown in the present study, involves several interplaying mechanisms. TNF-α is also a cytokine that may initiate the reperfusion-dependent cytokine cascade, induce the expression of adhesion molecules and chemokines, and promote leukocyte infiltration. Besides, TNF-α is also a potent proinflammatory cytokine that acts on myocardium cells to induce apoptosis and also propagates the inflammatory response during I/R injury [20]. Elevated levels of TNF-α in plasma have been implicated in various cardiovascular diseases, including heart failure and myocardial infarction. TNF-α is released locally from the storage site of myoplastic tissue mast cells during I/R. Additionally, it may also be released from cardiomyocytes after synthesis. In isolated rat hearts, I/R leads to the release of TNF-α and induces apoptosis of cardiomyocytes [21,22]. Numerous studies have shown myocyte apoptosis, or programmed cell death, may be central in cardiac I/R injury. Caspase-8, a proapoptotic protease, is activated during reperfusion, which finally leads to the degradation of nuclear DNA, resulting in cell death. Previous studies have shown that apoptosis correlates with the duration of ischemia and animal survival, and inhibition of apoptotic mediators, such as caspase-8, seems to be a rational therapeutic strategy to reduce the risks of preservation injury in the cardiac transplantation [23,24]. Furthermore, the complement system, as a part of the innate immune system, is one of the first responses to I/R injury, and it can determine the direction and magnitude of the subsequent responses. When C5 is cleaved by the classical or alternative pathway, C5 convertase, an effective anaphylatoxin C5A, is released. It mediates the entry of inflammatory mediators of neutrophils and lymphocyte lineages, leading to multiple effects, including cardiomyocyte apoptosis, during I/R injury. C5aR is present in myocardium cells of rats and up-regulated early after the onset of I/R injury. Such an increase in C5aR is accompanied by increased susceptibility of rat cardiomyocytes to C5a-induced apoptosis. It is necessary to block the complement deposition, such as C5a deposit to C5aR, in the transplanted heart to attenuate the I/R injury [25,26].

Neutrophils are important in host defense but may cause oxidative damage during inflammation. Neutrophils are thought to be closely related to the pathophysiology of heart transplant I/R injury [27,28]. After activation, neutrophils not only produce oxygen free radicals but also secrete MPO as part of the oxidation reaction. MPO is a heme enzyme secreted by activated neutrophils, which produces many oxidants, and is thought to play a key role in host defense and local tissue damage. [29,30]. In this study, TNF-α, caspase-8, and C5aR siRNA reduce I/R-induced myocardial tissue injury by inhibiting leukocyte infiltration and adherence. In addition, our study also showed that the levels of MDA in the heart tissue treated with high doses of TNF-α, caspase-8, and C5aR siRNA were significantly lower than those of the control group. Dose-dependency of siRNA and its effects on MDA production was parallel to the pathology manifestation of I/R injury in the cardiac isograft, the concentration of MDA was elevated as the extent of tissue injury was increased. Therefore, reducing lipid peroxidation may be one of the mechanisms of cardioprotective effects of intracoronary administration of TNF-α, caspase-8, and C5aR siRNA.

Gene silencing could effectively inhibit the protein in the heart long enough to cover this period of I/R injury. siRNA is a powerful tool that can be used to silence gene expression in mammalian cells. Thus, silencing myocardial TNF-α, caspase-8, and C5aR expression by siRNA delivery could be a promising and more advanced strategy for myocardial preservation. Both systemic and localized approaches have been used to deliver siRNA to the transplanted heart. Systemic siRNA delivery may be suitable in cases when the delivered siRNA silences a secreted factor that acts on neighboring or remote cells via a paracrine mechanism. Local administration of siRNA can greatly prevent local gene expression. For example, perfusing donor organs with an siRNA cocktail solution targeting complement 3, RelB, and Fas can induce renal gene silencing and prevent persistent cold I/R injury in kidneys during kidney transplantation [31]. Local exogenous gene delivery of foreign genes to the donor heart is currently administered primarily by intracoronary infusion, intramyocardial and intramyocardial injection, and pericardial perfusion. Because direct injection leads to uneven distribution of transgene expression and local inflammation, intracoronary infusion is more suitable for clinical applications [32]. In the clinical setting, the isolated donor organ is routinely perfused with a tissue preserving solution. This procedure could be combined with the administration of a therapeutic gene. In the current study, the uptake of small molecule siRNA by cardiac myocytes and endothelial cells was easy. In our research, a single intracoronary infusion of the cocktails is sufficient to transfect rat myocardial cell and can successfully suppress TNF-α, casepase-8, and C5aR expression. The efficacy of siRNA uptake in the cardiac isograft in the present study by intracoronary infusion siRNA under hypothermic conditions achieved the high myocardial transduction ratio. Thus, owning to the feasibility of design corresponding siRNA for human, this research provided possible approach to protect organs from I/R injury through intracoronary infusion of cocktails during harvesting donor organs in clinical setting [33,34].

Taken together, these studies indicate that TNF-α, caspase-8, and C5aR play an important role in the involvement of I/R injury during cardiac transplantation. As shown in the present study, the ability to transfer siRNA to heart grafts during cold storage has potentially profound clinical and research applications. Protection against I/R injury in rat cardiac transplantation could be achieved by means of intracoronary infusion of siRNA cocktail solution targeting TNF-α, caspase-8, and C5aR. In the long term, this approach may be part of a strategy to provide a milieu in which organ preservation may be achieved by the use of an siRNA cocktail solution.

## Abbreviations

C5aR: complement component 5a receptor
GAPDH: glyceraldehyde-3-phosphate dehydrogenase
H&E: hematoxylin-eosin staining
I/R: ischemia/reperfusion
MDA: malondialdehyde
MPO: myeloperoxidase
qRT-PCR: quantitative real-time PCR
siRNA: small interfering RNA
TNF-α: tumor necrosis factor-α
